# Ischemic Stroke Increases Protein Expression of the Folate Receptor and One-carbon Enzymes in Brain Tissue From Male and Female Patients

**DOI:** 10.1007/s12975-026-01479-w

**Published:** 2026-07-23

**Authors:** Petter Burrows, Himmat Dhillon, Gillian E. McDemott, Amanda Covaleski, Lilah Manfredi, Thomas G. Beach, Geidy E. Serrano, Nafisa M. Jadavji

**Affiliations:** 1 College of Veterinary Medicine, Midwestern University, Glendale, AZ USA; 2 College of Osteopathic Medicine, Midwestern University, Glendale, AZ USA; 3 Division of Molecular and Integrative Physiology, Department of Biomedical Sciences, School of Medicine, Southern Illinois University, Carbondale, Life Science III, room 2076 OR 2033, 1135 Lincoln Drive, Mail Code 6512, IL 62901 USA; 4 Department of Biomedical Sciences, Midwestern University, Glendale, AZ USA; 5 Banner Sun Health Research Institute, Sun City, AZ USA

**Keywords:** Ischemic stroke, Brain tissue, One Carbon Metabolism, Cerebral Cortex, Penumbra, Female, Male

## Abstract

Stroke is the second most common cause of death worldwide and predominantly affects individuals over 65 years old. Its prevalence is projected to increase in parallel with the aging global population. Nutrition is a modifiable risk factor for ischemic stroke. Folates, B-vitamins and choline play a central role in one-carbon metabolism (1 C), which is a key metabolic network that integrates nutritional signals with biosynthesis, redox homeostasis, epigenetics, regulation of cell proliferation, and stress resistance. Using preclinical models, our research group has previously shown that deficiencies in 1 C lead to worse stroke outcomes. However, the impact of ischemic stroke on 1 C enzymes in human brain tissue remains unexplored. The objective of this study is to investigate whether ischemic stroke contributes to a change in the levels of 1 C enzymes after ischemic stroke in male and female patients. Cortical brain tissue sections from ischemic stroke patients and controls were stained for enzymes involved in 1 C. All tissue was co-stained with neuronal nuclei (NeuN) and DAPI (4′,6-diamidino-2-phenylindole). The colocalization of all three markers was evaluated by two individuals who were masked to the experimental groups. Ischemic stroke increased neuronal levels of the folate receptor and 1 C enzymes, methylenetetrahydrofolate reductase (MTHFR), thymidylate synthase (TS) and serine hydroxy methyltransferase (SHMT). In male stroke brain tissue was observed to have increased levels of MTHFR, TS, and SHMT. Female brain tissue had increases in the folate receptor and TS. The results suggest that ischemic stroke leads to changes in neuronal levels of FR and 1 C enzymes levels in penumbra. Further clinical investigation is required to determine whether there is increase enzymatic activity and how 1 C is impacted in other cells within the brain, such as glial and endothelial cells.

**Graphical Abstract:**

**Figure:**
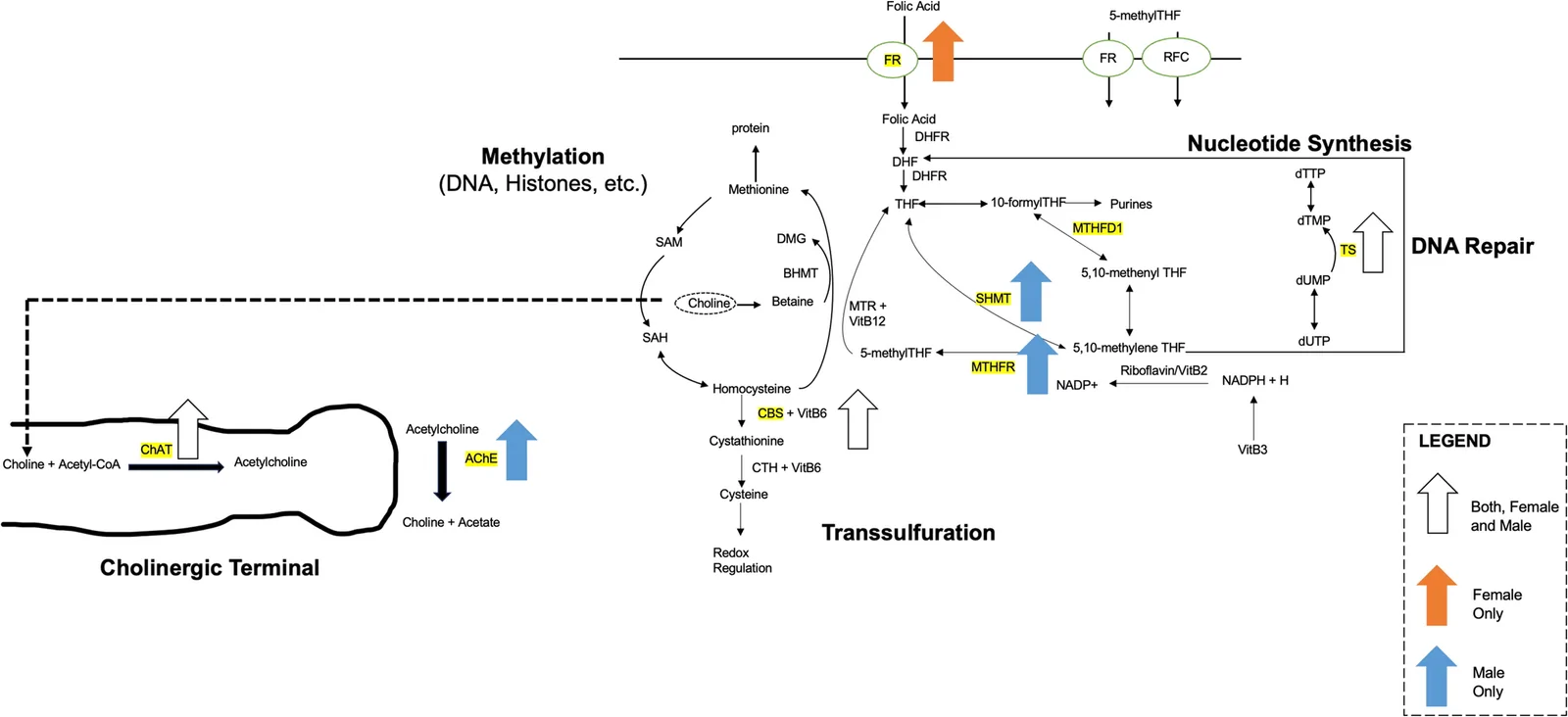

**Supplementary Information:**

The online version contains supplementary material available at https://doi.org/10.1007/s12975-026-01479-w.

## Introduction

Ischemic stroke ranks as the second most common cause of death worldwide and predominantly affects individuals aged 65 and older [1, 2]. With the changing demographics and aging of the global population, the incidence of medical emergencies like stroke is expected to increase [3]. Targeting risk factors of ischemic stroke, to reduce prevalence, is becoming more urgent. Nutrition is a modifiable risk factor for ischemic stroke [4, 5].

Elevated levels of homocysteine, a non-protein amino acid, are associated with an increased risk of cardiovascular disease, such as ischemic stroke [6, 7]. B-vitamins including folic acid (vitamin B9) and vitamin B12 play crucial roles in reducing levels of homocysteine, through the one-carbon (1 C) metabolism pathway [8]. In older individuals, decreased absorption of B-vitamins from the gastrointestinal tract can predispose them to ischemic stroke [9]. In addition to cofactor deficiency, the enzymes that work to metabolize homocysteine could contain inherent genetic changes. For example, methylenetetrahydrofolate reductase (MTHFR) is a critical enzyme in methylating homocysteine thereby studies have characterized the effects of a 677 C→ T genetic polymorphism in this enzyme with development of vascular disease [10]. As a key metabolic network 1 C integrates nutritional signals with biosynthesis, redox homeostasis, and epigenetics, it plays essential roles in regulation of cell proliferation, and stress resistance [11]. The onset of ischemic stroke impacts many of these cellular processes.

Ischemic stroke occurs more frequently in men than in women of the same age. However, in total, women experience more strokes than men because women have a longer life expectancy and a much higher incidence of stroke at older ages [12]. Stroke incidence in women increases significantly after menopause, suggesting that hormonal changes might play a role. The prevalence and incidence of stroke increases in postmenopausal women, leading to higher mortality rates and case fatality rates for older women, 60% of stroke fatalities are attributed to women [13]. It is believed premenopausal women are protected from stroke due to mechanisms related to gendered hormones. Estrogen appears to be neuroprotective, enhancing blood flow by reducing vascular reactivity, while testosterone has opposing effects. It is also important to consider that women tend to live longer, which affects their stroke statistics [12]. There is still much to be discovered about the variations in stroke between men and women.

The current literature demonstrates elevated homocysteine levels with an increased risk of ischemic stroke [6, 7, 14, 15]. Our work in preclinical models has demonstrated that dietary [16–21] or genetic [10, 22] deficiencies in 1 C lead to worse outcomes for animals post-stroke. The impact on 1 C post-stroke remains relatively undefined in human ischemic stroke patients. The aim of this study was to investigate whether ischemic stroke impacts neuronal protein expression of the folate receptor and enzymes involved in 1 C using patient samples in the post-mortem brain tissue of male and female patients.

## Methods

This study was conducted in accordance with the Declaration of Helsinki and was determined to be Internal Review Board (IRB) exempt as per the Midwestern University IRB Committee. Cortical human brain tissue was obtained from Banner Sun Health Research Institute Brain and Body Donation Program [23]. Informed consent was obtained from all individual participants included in the study. Cortical slide mounted brain tissue sections from the penumbra of female and male with acute ischemic stroke and healthy control subjects was obtained. The penumbra was defined by reduction of neuronal density [24]. All stroke patients had cerebral ischemic strokes as confirmed by pathological analysis using H & E Staining, quantification of infarct volume is listed in Table 1. Furthermore, details of patients including age and comorbidities are listed in Table 1.

**Table 1:** Patient sample demographics and comorbidities

	Control		Stroke	
Age	Male	Female	Male	Female
90+	1/7 (14%)	0	5/9 (55%)	5/7 (71%)
80–90	1/7 (14%)	0	2/9 (22%)	2/7 (29%)
75–80	1/7 (14%)	4/7 (57%)	1/9 (11%)	0
70–75	4/7 (57%)	3/7 (43%)	1/9 (11%)	0
**Comorbidities**				
CAA	-	-	8/9 (88%)	5/7 (71%)
MCI	-	-	1/9 (11%)	1/7 (14%)
MSA	-	-	1/9 (11%)	0
PD	-	-	0	0
VaD	-	-	3/9 (33%)	1/7 (14%)
Average Infarct Volume (cm^3)	-	-	18.4	8.28

*CAA* Cerebral amyloid angiopathy, *MCI* Mild Cognitive Impairment, *MSA* Multi-System Atrophy, *PD* Parkinson’s disease, *VaD* Vascular dementia

### Immunofluorescence Experiments

**Fig. 1:**
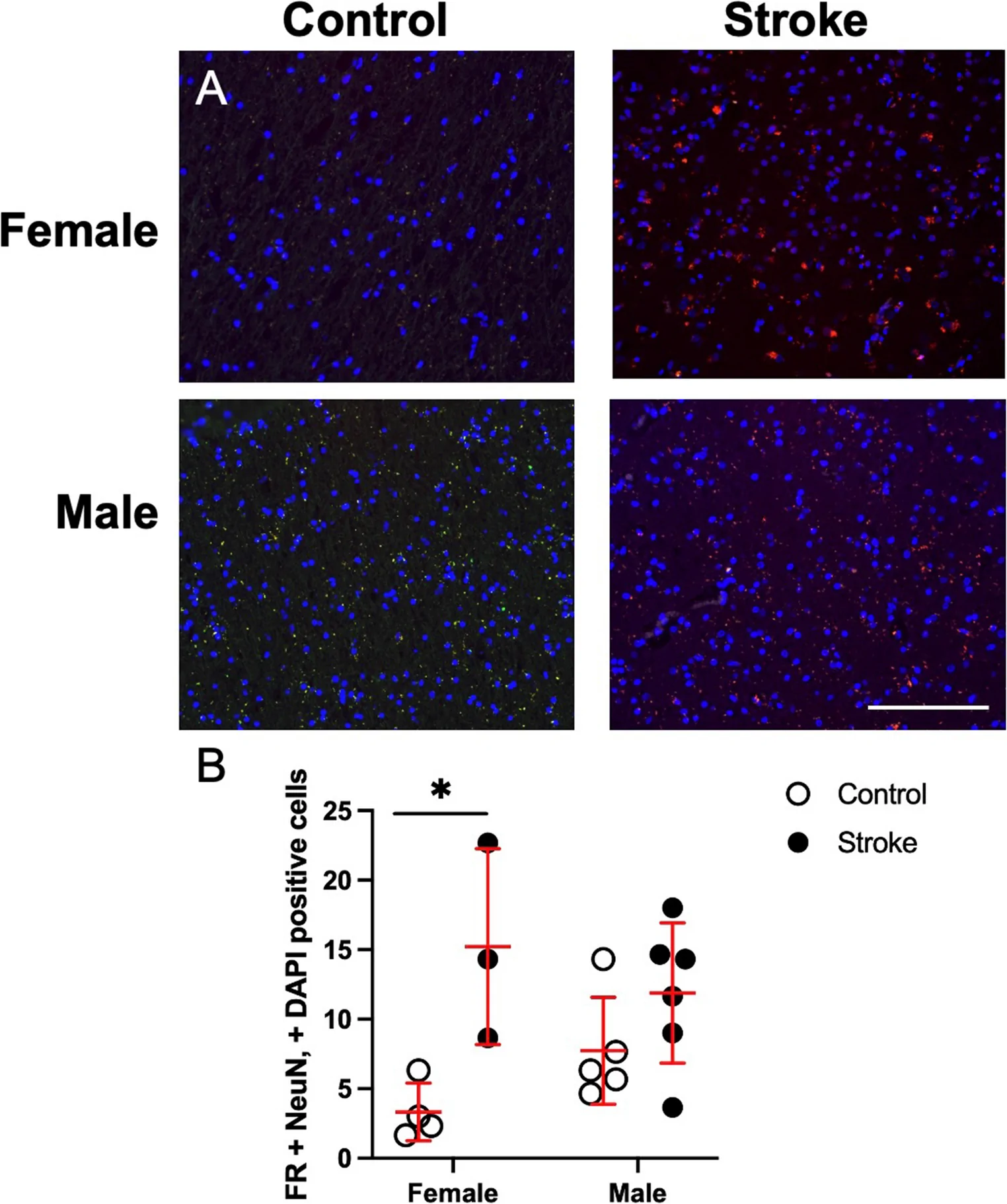
Representative images of the levels of folate receptor (FR; **A**), co-stained with neuronal nuclei (NeuN) and DAPI in brain tissue of healthy aged controls and ischemic stroke patients. Quantification of FR (**B**) using a semi-quantitative method. Data represents 3 to 6 patients per group. Sample IDs, female control: 3428, 4636,2076, 2082, 2080; male control: 2083, 2077, 4638, 3421; female stroke: 2131, 1252,1240, 906; male stroke: 1532, 1521, 0940, 0112, 1421, 1577. 2-way ANOVA revealed difference between control and stroke patients of FR levels. * *p* < 0.05, Tukey’s pairwise comparison between control and stroke female patients. Scale bar at 50 μm

To investigate neuronal 1 C enzyme levels, immunofluorescence experiments of ischemic stroke and control brain tissue was performed (Fig. 1). For each antibody we used an *n* = 3 samples per group, as determined by a priori sample size calculations using G*3 Power [25]. The observed effect size was d = 1.25, alpha level was 0.05, power was set to 80% [26]. If there was variability staining, the sample size was increased to 4 or 5.

Tissue sections were deparaffinized using xylene and ethanol and blocked with 5% normal goat serum (Jackson Immuno Research Lab, PA, US, catalog: NC966007) diluted in 0.5% Triton-X (Millipore Sigma, MA, USA, Catalog: 64846650ML). Slides were then incubated with primary antibody diluted in 5% normal goal serum with 0.5% Triton-X overnight at 4 °C. The following primary antibodies were used to identify the specific protein in question for a given sample: FR⍺ (1:100, Thermo Fisher, Waltham, MA, USA, RRID: AB_2609390), MTHFR (1:100, AbCam, Boston, MA, USA, RRID: AB_2893493), SHMT1 (1:100, Cell Signaling, Danvers, MA, USA, catalog # 80715), TS (1:100, Cell Signaling, Danvers, MA, USA, catalog # 9045), ChAT (1:100, Millipore Sigma, Darmstadt, Germany, RRID: AB_2079751), AChE (1:100, Millipore Sigma, Darmstadt, Germany, RRID: AB_10602654) were used. All brain sections were stained with a marker for neuronal nuclei, NeuN (1:200, AbCam, Waltham, MA, USA, RRID: AB_10711040).

Brain sections were incubated with secondary antibodies Alexa Fluor 488 or 555 (1:200, Cell Signaling Technologies) the following day at room temperature for 2 h then stained with 4′, 6-diamidino-2phenylindole (DAPI, 1:10,000). Prior to beginning experimental studies, we stained tissue with secondary antibodies to determine whether non-specific binding was present. Both secondary antibodies did not show any non-specific binding. Microscope slides were cover slipped with fluromount and stored at 4℃ until analysis.

### Immunofluorescence Analysis

The staining was visualized using the Revolve microscope (Echo, San Diego, CA, USA) and all images were collected at the magnification of 200×. We used a semi-quantitative method to assess 1 C protein levels within ischemic stroke and control brain tissue. Specifically, colocalization of the primary antibody with NeuN and DAPI-labeled neurons was counted and averaged across three sections per primary antibody per subject. Using FIJI (NIH) [27] cells were distinguished from debris by identifying a clear cell shape and intact nuclei (indicated by DAPI and NeuN) under the microscope. All cell counts were conducted by at least two individuals blinded to treatment groups.

### Statistics

The inclusion criteria for this study were whether the sample was able to be stained for DAPI and NeuN. If the sample could not be stained with both markers, it was not included. GraphPad Prism 10.5.0 was used to immunofluorescence staining quantification. In GraphPad Prism a D’Agostino-Pearson normality test was performed prior to two-way ANOVA analysis when comparing the mean measurement of both gender and group (control or stroke). Significant main effects of two-way ANOVAs were followed up with Tukey’s post-hoc test to adjust for multiple comparisons. All data are presented as mean *±* standard deviation (SD). Statistical tests were performed using a significance level of 0.05.

## Results

### Increased Levels of Folate Receptor in Female Stroke Patients

Using immunofluorescence, we stained brain tissue from healthy controls and stroke patients for the folate receptor (FR), which is a cell surface protein that binds and brings vitamin B9 (folate) and folic acid into the cell. Representative images of folate receptor neuronal staining are shown in Fig. 1A. Ischemic stroke impacted neuronal folate receptor levels in cortical tissue (Fig. 1B, F(_1,14_) = 12.72, *p* = 0.0031). Females stroke patients had more expression of the folate receptor compared to healthy controls (*p* = 0.021).

### Male Stroke Patients Have Increased Levels of Methylenetetrahydrofolate Reductase (MTHFR)

**Fig. 2:**
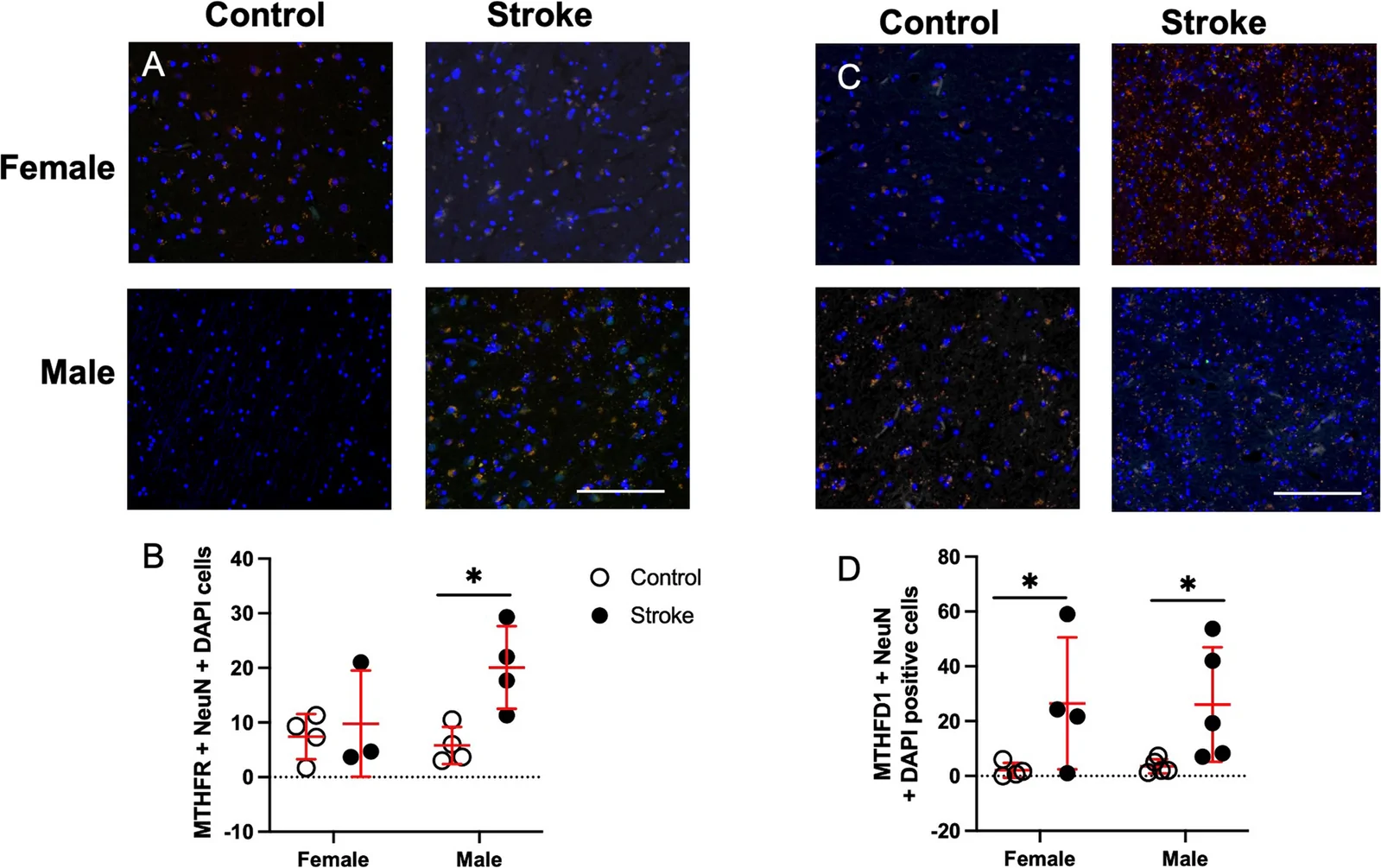
Representative images of the levels of methylenetetrahydrofolate reductase (MTHFR; **A**), and methylenetetrahydrofolate dehydrogenase 1 (MTHFD1; **C**) co-stained with neuronal nuclei (NeuN) and DAPI in brain tissue of healthy aged controls and ischemic stroke patients. Quantification of MTHFR (**B**), and MTHD1 (**D**) using a semi-quantitative method. Data represents 3 to 5 patients per group. Sample IDs, female control: 4636, 3428, 2079, 2081, 2075; male control: 3421, 4638, 1926, 4204; female stroke: 1194, 0426, 1958, 2131, 1252; male stroke: 1421, 1577, 1240, 2036, 1517. 2-way ANOVA revealed difference between control and stroke patients of MTHFR, and MTHFD1 levels. * *p* < 0.05, Tukey’s pairwise comparison between control and stroke patients. Scale bar at 50 μm

Methylenetetrahydrofolate reductase (MTHFR) is a key enzyme in 1 C, as it converts vitamin B9 and folic acid into the active form to be used and promotes the conversion of homocysteine into methionine [28]. Representative images of neuronal MTHFR staining are shown in Fig. 2A. Ischemic stroke affected levels of neuronal MTHFR in cortical brain tissue (Fig. 2B, F(_1,11_) = 12.72, *p* = 0.029). Male ischemic stroke patients had higher levels of MTHFR compared to healthy controls (*p* = 0.038).

### Methylenetetrahydrofolate Dehydrogenase (MTHFD1) Elevated in Female and Male Stroke Patients

MTHFD1 is a critical gene encoding a trifunctional enzyme that converts folate into essential components for DNA synthesis, repair, and methylation [29]. Representative images of neuronal MTHFD1 staining are shown in Fig. 2C. Ischemic stroke affected levels of neuronal MTHFD1 levels in cortical brain tissue (Fig. 2D, F (_1,14_) = 9.74, *p* = 0.0075). Both female (*p* = 0.0471) and male (*p* = 0.0413) had increased levels of neuronal MTHFD1 compared to healthy controls.

### Thymidylate Synthase (TS) Levels Elevated in Female and Male Patients

**Fig. 3:**
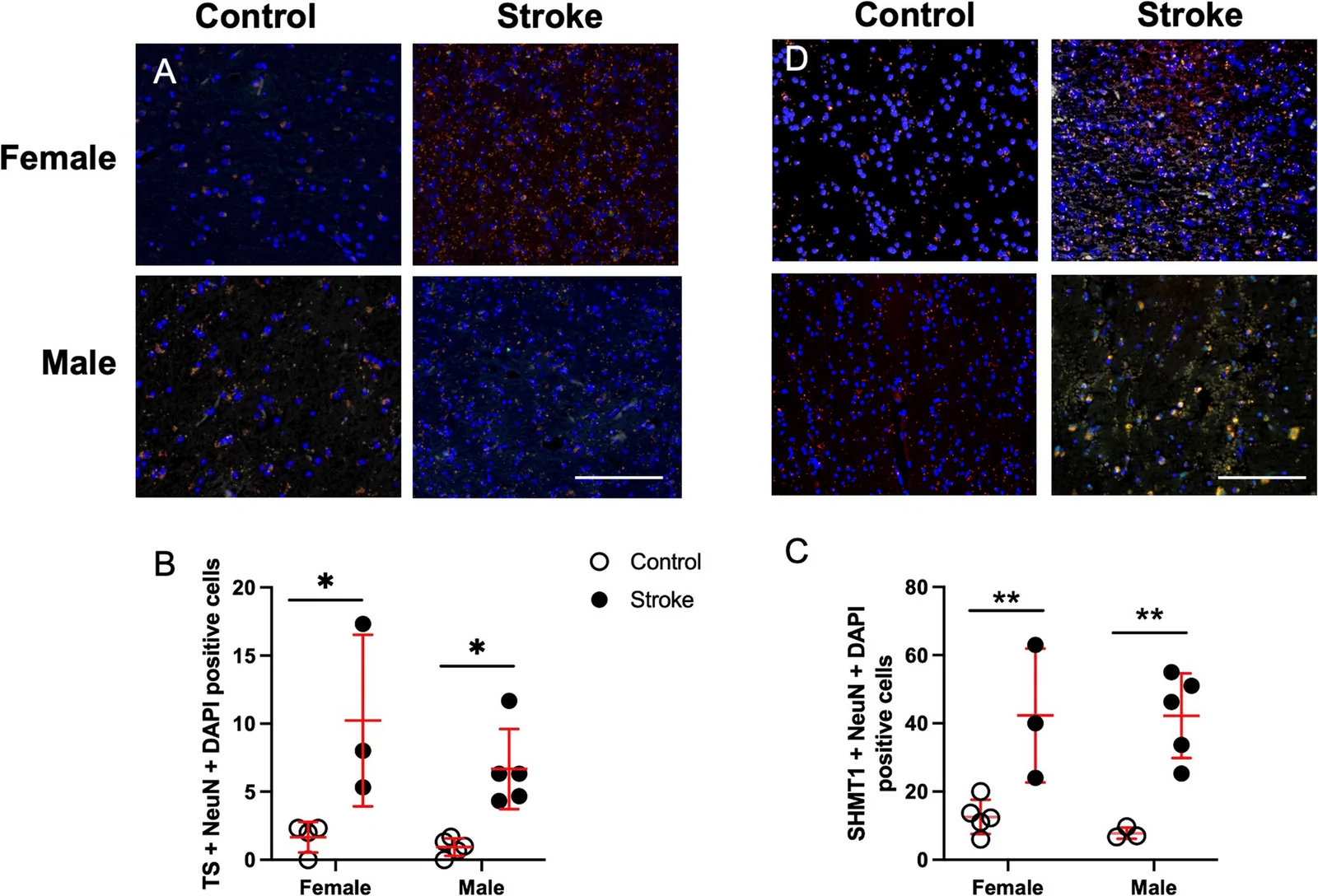
Representative images of the levels of thymidylate synthase (TS; **A**) and serine hydroxymethyltransferase (SHMT; **B**), co-stained with neuronal nuclei (NeuN) and DAPI in brain tissue of healthy aged controls and ischemic stroke patients. Quantification of TS (**B**) and SHMT (**D**) using a semi-quantitative method. Data represents 3 to 5 patients per group. Sample IDs, female control: 2080, 2081, 3428; male control: 2079,2083,3421, 4204; female stroke: 1240, 1252, 2131; male stroke: 2036, 1958,1926, 1421. 2-way ANOVA revealed difference between control and stroke patients of TS and SHMT levels. * *p* < 0.05, ** *p* < 0.01, Tukey’s pairwise comparison between male or female control and stroke patients. Scale bar at 50 μm

We measured the impact of stroke on DNA biosynthesis and repair. Using TS which is involved in removal of uracil from DNA [30]. Representative images of neuronal TS staining are shown in Fig. 3A. Ischemic stroke impacted levels of neuronal TS in cortical brain tissue (Fig. 3B, F (_1,13_) = 22.59, *p* = 0.0004). Both female (*p* = 0.013) and male (*p* = 0.0453) ischemic stroke patients had higher levels of neuronal TS in brain tissue.

### Serine Hydroxymethyltransferase (SHMT) 1 Levels Increased in Females and Males

SHMT is an enzyme that reversibly converts serine and tetrahydrofolate (THF) into glycine and 5,10-methylene-THF. It plays a central role in one-carbon metabolism, supporting nucleotide synthesis, amino-acid balance, and redox control [31]. Representative images of neuronal SHMT1 staining are shown in Fig. 3C. Ischemic stroke impacted levels of neuronal SHMT1 in cortical brain tissue (Fig. 3D, F (_1,12_) = 9.15, *p* = 0.0085). Both female (*p* = 0.003) and male (*p* = 0.0012) ischemic stroke patients had higher levels of neuronal SHMT1 in brain tissue.

### Choline Metabolism Impacted By Stroke and Gender

**Fig. 4:**
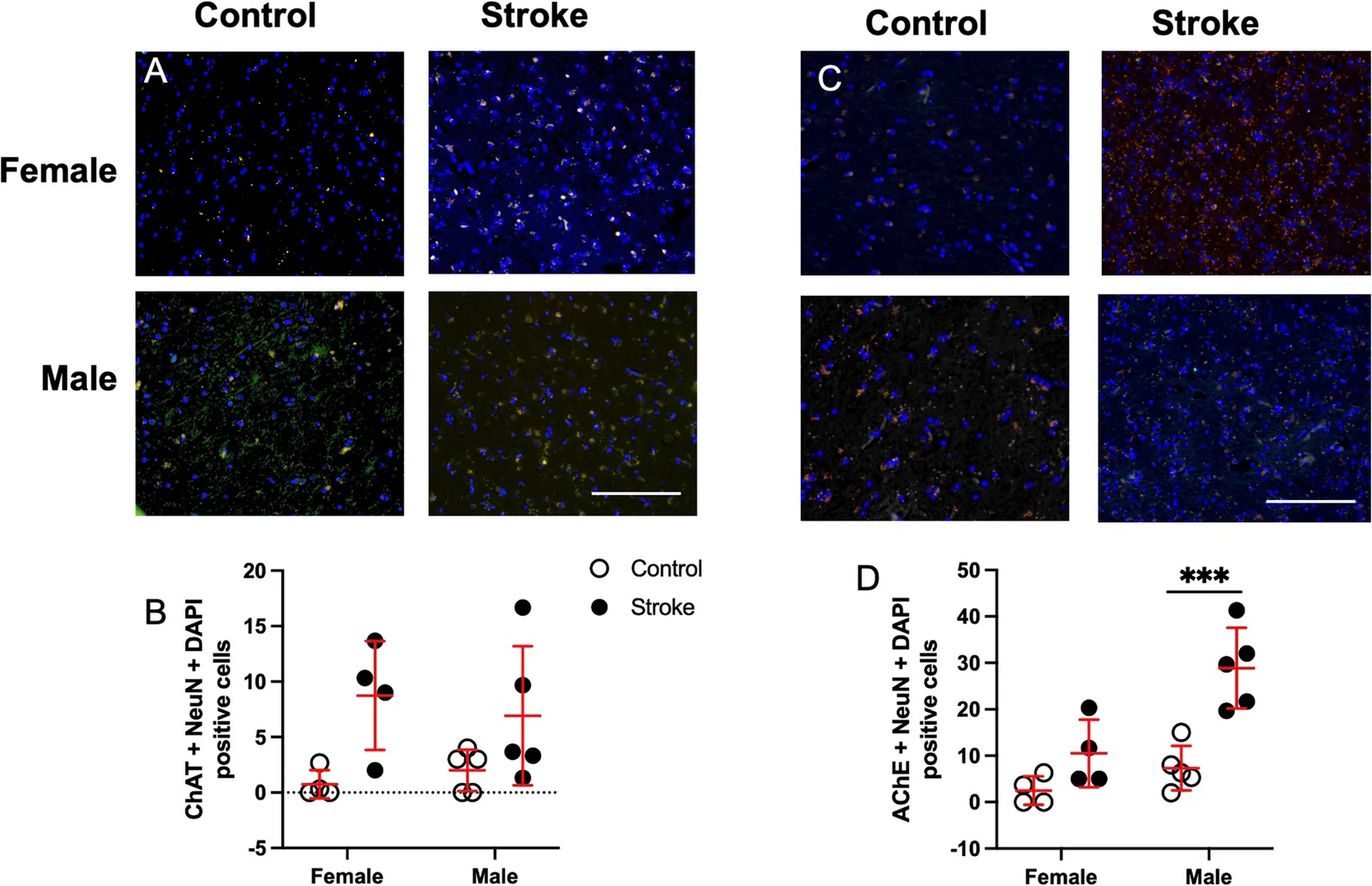
Representative images of the levels of choline acetyltransferase (ChAT; **A**), and acetylcholinesterase (AChE; **B**), co-stained with neuronal nuclei (NeuN) and DAPI in brain tissue of healthy aged controls and ischemic stroke patients. Quantification of ChAT (**B**) amd AChE (**D**) using a semi-quantitative method. Data represents 3 to 5 patients per group. Sample IDs, female control: 2075, 2076, 2082, 4636; male control: 2077, 2078, 3421, 4204, 4638; female stroke:426,906, 1194, 1912; male stroke: 112, 1521, 1532, 1926, 2036. 2-way ANOVA revealed difference between control and stroke patients of ChAT and AChE levels. *** *p* < 0.001, Tukey’s pairwise comparison between control and stroke male or female patient. Scale bar at 50 μm

**Fig. 5:**
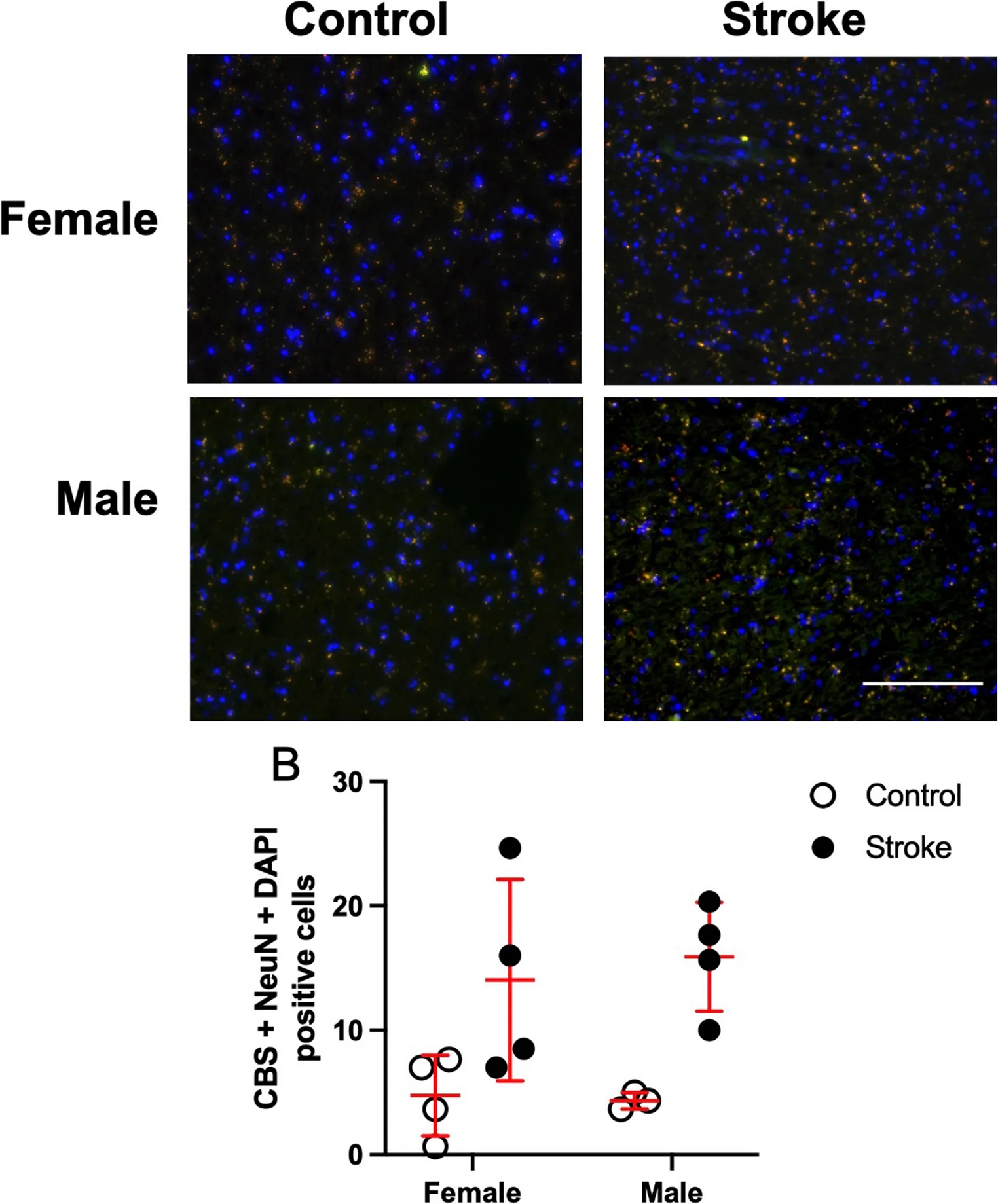
Representative images of the levels of Cystathionine-β-synthase (CBS; **A**) co-stained with neuronal nuclei (NeuN) and DAPI in brain tissue of healthy aged controls and ischemic stroke patients. Quantification of CBS (**B**) using a semi-quantitative method. Data represents 3 to 5 patients per group. Sample IDs, female control: 2080, 2081, 4636; male control: 4204, 3421, 2079; female stroke: 1252, 1912, 2131; male stroke: 940, 1421, 1532, 1577. 2-way ANOVA revealed difference between control and stroke patients of CBS levels. Scale bar at 50 μm

Choline metabolism is tightly linked to 1 C, as choline can donate methyl groups to remethylate homocysteine to methionine. Our previous work has shown that deficiencies in 1 C are linked impact choline metabolism [32]. Representative images of neuronal ChAT staining are shown in Fig. 4A. Ischemic stroke affected levels of neuronal ChAT levels in cortical brain tissue (Fig. 4B, F(_1,14_) = 10.47, *p* = 0.0060). There was also a gender difference between female and male patients (F(_1,14_) = 5.39, *p* = 0.036). There was no interaction between gender and stroke (*p* = 0.32) (Fig. 5).

Representative images of neuronal AChE staining are shown in Fig. 4C. Ischemic stroke affected levels of neuronal AChE levels in cortical brain tissue (Fig. 5, F(_1,14_) = 23.31, *p* = 0.0003). There was also a gender difference between female and male patients (F(_1,14_) = 14.39, *p* = 0.0020) and an interaction between gender and health status (F(_1,14_) = 4.894, *p* = 0.0441). Male ischemic stroke patients had higher levels of neuronal AChE compared to healthy controls (*p* = 0.0006).

### Ischemic Stroke Increases Levels of Cystathionine-β-synthase (CBS)

CBS enables the breakdown of homocysteine and contributes to the synthesis of cysteine and glutathione, essential for fighting oxidative stress [33]. Representative images of neuronal CBS staining are shown in Fig. 4A. Ischemic stroke affected levels of neuronal CBS levels in cortical brain tissue (Fig. 4B, F(_1,11_) = 15.41, *p* = 0.0024). There was no impact of gender (*p* = 0.90) and interaction between gender and stroke (*p* = 0.77) (Fig. 5).

## Discussion

One-carbon (1 C) metabolism plays a crucial role in various physiological processes, including biosynthesis of nucleotides, amino acid homeostasis, epigenetic maintenance, and redox protection. Additionally, current literature does indicate that elevated levels of homocysteine are a risk factor for ischemic stroke [34, 35]. However, data investigating 1 C enzyme protein levels between healthy and stroke-affected human brain tissue are lacking, as well as whether there are gender differences. Our study aimed to determine whether ischemic stroke alters 1 C enzyme levels by analyzing post-mortem cortical penumbra brain tissue from both male and female patients. All 1 C enzymes we measured were increased in stroke patients compared to healthy controls. Interestingly, we observed differences between female and male tissue. Notably, our results indicated female ischemic stroke patients had elevated expression of the folate receptor when compared to healthy female control. This may suggest that there is an increased demand of folates in the brain post-stroke in females, however investigating other cell types in the brain as well as enzymes levels is needed. Male ischemic stroke patients had elevated MTHFR and AChE levels compared to healthy controls. Both female and male stroke patients showed significantly elevated levels of MTHFD1, SHMT, and TS compared to healthy controls. Our data demonstrates that there is an impact of gender on 1 C enzyme levels following ischemic stroke in cortical brain tissue. These observed alterations in 1 C enzyme expression may correspond with the established gender-specific differences in stroke presentation, risk factors, and metabolic responses post-stroke.

Between genders, presenting symptoms of stroke differ, with women reporting atypical stroke symptoms at a higher prevalence as compared to men [36–38].Women report headaches, consciousness or mental status changes, and coma, while men typically report paresis/hemiparesis and focal visual disturbances. Women usually present with more severe stroke symptoms and are 4 to 5 years older than men at onset [36]. Further, young men have a much higher risk of stroke compared to premenopausal women. However, this trend flips after menopause, leading to the incidence of stroke in older women being twice that of older men. A reversal often attributed to the loss of hormone production from the ovaries that coincides with menopause [37]. Congruently, an increased risk of stroke has been indicated in women with later age at menarche, > 15 years of age, and a short reproductive life span, ≤ 36 years of age. Overall, a short reproductive life span for women is associated with an increased risk of nonfatal cardiovascular disease events, such as stroke, in midlife [39].

Gender differences in metabolism after ischemic stroke are increasingly apparent but remain unexplored. Limited studies suggest that females exhibit greater changes in amino acid and lipid metabolism after stroke, including higher levels of neurotoxic kynurenine metabolites, which are linked to worse post-stroke outcomes. Further, one preclinical study showed that Huang-Lian-Jie-Du decoction improved neurological function after stroke in both male and female rat models, with greater benefit in females, potentially due to gender-specific differences in metabolites linked to oxidative stress regulation [40]. These findings begin to highlight how gender differences in 1 C metabolism mirror the differing outcomes seen post-stroke between genders. Gender differences in 1 C metabolism have been reported in healthy patients, showing that female shave lower plasma homocysteine levels and *S*-adenosylmethionine, but higher levels of choline and betaine. These changes have been reported in the relation to pregnancy and fetal development [41]. Our study did not include measurements of homocysteine, and the female patients were well past their reproductive age. There might be other factors such as levels of testosterone [42] and potential cancer status [43] might be driving the sex differences. The present study has added to this body of literature demonstrating gender differences in 1 C enzyme levels in elderly male and female stroke patients.

One-carbon metabolism with folate support is essential for nucleotide synthesis, homocysteine remethylation, and numerous biosynthetic processes critical for growth and development. Thus, disruptions in folate metabolism can impair cellular proliferation, influence epigenetic regulation, and increase the risk of congenital disabilities or disease [35]. Studies show men exhibit higher plasma concentrations of choline, betaine, and homocysteine and an increased likelihood of folate deficiency. In the present study we report increased levels of neuronal levels of AChE in male stroke patients and increased levels of ChAT in both stroke females and males. AChE is an enzyme involved in breaking down acetylcholine at the synapse and ChAT is an enzyme that synthesizes it. Previous work from our group has shown that choline compensates in the brain during elevated levels of homocysteine, as a result of deficiencies in folate metabolism, either genetic [32] or dietary [44]. It is well documented that aging results in increased levels of homocysteine [45], indicating the need for increased levels of AChE and ChAT in the entirety of the aging population.

The importance of 1 C metabolism can also be seen by the increases in MTHFD1, SHMT, TS, and CBS in both stroke females and males in the present study. MTHFD1 encodes a key enzyme in folate-mediated 1 C metabolism, essential for nucleotide synthesis and methylation, with disruptions linked to metabolic imbalance and cognitive deficits [46]. Its increase post-stroke likely reflects a metabolic flux response post-stroke, aimed at boosting one-carbon metabolism to support DNA repair, methylation, and reduce harmful homocysteine through enhanced folate-driven reactions.

SHMT is a vitamin B_6_-dependent enzyme that catalyzes the reversible conversion of serine and tetrahydrofolate into glycine and 5,10-methylene‑THF, supplying one-carbon units essential for nucleotide synthesis, methylation, and redox balance [47]. Its increase in the present study aligns with the literature that found ischemic stroke patients exhibit significantly higher SHMT1 promoter methylation compared to healthy controls, which is strongly associated with elevated plasma homocysteine levels. This suggests that stroke triggers epigenetic changes that suppress SHMT1 expression, prompting metabolic flux to normalize folate-dependent one-carbon metabolism and manage toxic homocysteine accumulation [48].

TS catalyzes the conversion of deoxyuridine monophosphate (dUMP) to deoxythymidine monophosphate (dTMP) using 5,10‑methylene‑THF as a methyl donor, which provides the only *de novo* source of thymine nucleotides essential for DNA synthesis and repair [49]. The findings in the present study conform with accepted idea that after ischemic stroke, specific TS gene polymorphisms are more common and are associated with increased TS expression, which likely enhances DNA repair and one-carbon metabolism to counteract tissue damage and elevated homocysteine levels [50]. Thus, stroke patients may exhibit upregulated TS activity as a metabolic flux to vascular injury and metabolic stress.

CBS catalyzes the first and rate-limiting step of the transsulfuration pathway, combining homocysteine and serine to form cystathionine. This is a crucial process for detoxifying neurotoxic homocysteine and producing cysteine for glutathione synthesis to support antioxidant defenses. After ischemic stroke, elevated homocysteine becomes neurotoxic and pro-inflammatory. As described in the literature and seen in the present study, CBS is upregulated post-stroke to convert homocysteine into cystathionine, aiding in detoxification and supporting antioxidant defense via glutathione synthesis, thus serving as a crucial compensatory mechanism against oxidative stress in the damaged brain [35]. These changes in 1 C metabolism highlight the effects and underscore the complications of ischemic stroke seen in stroke patients.

In women estrogen upregulates phosphatidylethanolamine N-methyltransferase, enhancing phosphatidylcholine synthesis and influencing 1 C flux. These differences in metabolism and hormones contribute to gender-specific methylation patterns [51]. In men, choline and homocysteine levels are associated with specific epigenetic modification that regulates DNA repair and oncogenic risk [51, 52]. Meanwhile, choline and vitamin B12 correlate with protective epigenetic modification essential for genome stability and hematopoiesis in women [32]. Further, mathematical modeling indicates estrogen-driven upregulation of phosphatidylethanolamine methyltransferase in women significantly elevates choline and betaine concentrations. This increase enriches tissue-level activation of cystathionine β-synthase, which is the primary enzyme responsible for homocysteine clearance. As a result, females exhibit lower plasma homocysteine levels compared to males [53]. Overall, this previous work and our study highlight how gender-specific regulation of 1 C metabolism shapes not only nutrient status, but also disease risk, developmental outcomes, and epigenetic programming.

One-carbon (1 C) metabolism plays an important role in the generation of *S*-adenosylmethionine, a global methyl donor [54]. Changes in DNA methylation after ischemic stroke have been reported in preclinical models and patients ranging from hyper to hypomethylation [55, 56]. This data suggests that DNA methylation has a role for inducing stroke pathologies and/or managing recovery mechanisms after disease onset. Changes in methylation of pathophysiological events after stroke including excitotoxicity, oxidative stress, mitochondrial dysfunction, blood-brain barrier (BBB) disruption, apoptosis and inflammation have been reported [55, 57]. Furthermore, there have been recovery changes including neurogenesis, angiogenesis, gliogenesis, axon growth, and synaptic plasticity [55]. Understanding the implication of methylation pattern changes post-stroke is an area that needs more attention.

In the present study, stroke females had significantly increased levels of folate receptor possibly coinciding with the neuroprotective nature of estrogen. Estrogen does this by triggering the rapid retention of kinases like PI3K/Akt and ERK, leading to enhanced cell survival signaling and reduced oxidative stress in peri‑infarct tissues. These same signaling pathways are also involved in upregulating nutrient transport systems like folate metabolism during repair from damage, i.e., ischemic stroke [58]. Further, these gender differences in 1 C metabolism may also explain the significantly increased levels of MTHFR found in males. The literature suggests that testosterone boosts MTHFR activity, while estradiol suppresses it, indicating that males inherently have higher MTHFR activity due to their androgen levels [53]. This suggests that after a stroke, male patients are more likely to upregulate MTHFR in response to ischemic stress and homocysteine accumulation. In contrast, females, influenced by estrogen, maintain more stable MTHFR levels.

This is the first study to investigate neuronal protein levels of 1 C enzymes after ischemic stroke in penumbra cortical tissue of aged patients. Our study was limited to the cerebral cortex as well as measuring 1 C enzyme levels only in neuronal cells. Expanding to other areas of the brain and investigating the impact of stroke on other cell types (e.g., glial and endothelial cells) is needed to gain a comprehensive understanding of the impact ischemic stroke has on 1 C enzyme levels in the brain. Neuronal analysis is a limitation of our study. Additionally, it is important to note that stroke patients had more CAA prevalence and this could be contributing to the changes in 1 C enzymes and FR [59]. Additionally, there was a large age gap between stroke and control patients which may serve as a confounding factor [60].

In terms of next steps, investigating levels of RNA would provide vital information, unfortunately, we were not certain about the quality of samples. Furthermore, expanding data collection to include enzyme activity and in blood homocysteine levels, as well as other 1 C metabolites would add to the data set. Gaining access to functional scores from patients and details of sample collection are needed to gain a better understanding. A comprehensive understanding of how ischemic stroke impacts and interactions with 1 C will enable future therapeutic developments with a precision medicine focus. Lastly, changes in DNA methylation post-stroke and linking it to 1 C is a stimulating opportunity for investigation.

## Supplementary Information

Below is the link to the electronic supplementary material.ESM 1DOCX (16.8 KB)

## Data Availability

No datasets were generated or analysed during the current study.
