# Supplementary material for: Ischemic Stroke Increases Protein Expression of the Folate Receptor and One-carbon Enzymes in Brain Tissue From Male and Female Patients
Source: Transl Stroke Res. 2026 Jul 23;17(4):87. doi: 10.1007/s12975-026-01479-w (PMC13395894; doi:10.1007/s12975-026-01479-w)
Supplement: Supplementary file 1 — DOCX (16.8 KB) [file 12975_2026_1479_MOESM1_ESM.docx]

Supplementary Data

**Table 1.** Mean + Standard deviation of all semi-quantitative immunofluorescence analysis.

|  |  | **Female** |  |  |  | **Male** |  |
| --- | --- | --- | --- | --- | --- | --- | --- |
| **Antibody** | **Control** |  | **Stroke** |  | **Control** |  | **Stroke** |
|  |  |  |  |  |  |  |  |
| FR | 3.33 + 2.07 |  | 15.22 + 7.04 |  | 7.73 + 3.85 |  | 11.89 + 5.04 |
|  |  |  |  |  |  |  |  |
| MTHFR | 7.42 + 4.19 |  | 9.78 + 9.73 |  | 5.80 + 3.39 |  | 20.08 + 7.56 |
|  |  |  |  |  |  |  |  |
| MTHFD1 | 2.81 + 2.70 |  | 26.5 + 24.05 |  | 3.53 + 2.56 |  | 26.07 + 20.85 |
|  |  |  |  |  |  |  |  |
| TS | 1.67 + 1.12 |  | 10.22 + 6.30 |  | 0.93 + 0.64 |  | 6.67 + 2.94 |
|  |  |  |  |  |  |  |  |
| SHMT | 12.60 + 5.05 |  | 42.33 + 19.6 |  | 7.78 + 1.64 |  | 42.23 + 12.41 |
|  |  |  |  |  |  |  |  |
| ChAT | 0.75 + 1.29 |  | 8.75 + 4.91 |  | 2.00 + 1.87 |  | 6.93 + 6.27 |
|  |  |  |  |  |  |  |  |
| AChE | 2.50 + 3.81 |  | 10.50 + 7.27 |  | 7.33 + 4.81 |  | 28.87 + 8.69 |
|  |  |  |  |  |  |  |  |
| CBS | 4.75 + 3.24 |  | 14.04 + 8.10 |  | 4.33 + 0.67 |  | 15.92 + 4.38 |
